# Progressive Multifocal Leukoencephalopathy: A Rare Cause of Altered Mentation

**DOI:** 10.7759/cureus.104554

**Published:** 2026-03-02

**Authors:** Arcole Brandon, Shabnam Eghbali, Suzanne Sharpton

**Affiliations:** 1 Department of Medicine, Vanderbilt University Medical Center, Nashville, USA; 2 Department of Gastroenterology, Hepatology, and Nutrition, Vanderbilt University Medical Center, Nashville, USA

**Keywords:** cirrhosis, demyelinating diseases, encephalopathy, immunosuppression, progressive multifocal leukoencephalopathy, sarcoidosis, sjogren's syndrome

## Abstract

Progressive multifocal leukoencephalopathy (PML) is a rare demyelinating neurologic disease caused by the John Cunningham virus (JCV). Most affected patients have human immunodeficiency virus (HIV), but medications are also a risk factor. It can be difficult to diagnose PML as it can present with a broad range of symptoms, especially if other conditions are present that also contribute to encephalopathy. We report a case of a patient who developed PML from medications rarely associated with PML in the setting of multiple comorbid medical conditions that contributed to diagnostic delay. A 63-year-old female patient presented with subacute, intermittent encephalopathy, weakness, and dysphagia. She had a known history of cirrhosis, Sjogren’s syndrome, and sarcoidosis, for which she was on chronic azathioprine and prednisone. Initial workup showed laboratory and exam findings consistent with hepatic encephalopathy (HE) and cystitis. Despite appropriate treatment, her condition worsened, and magnetic resonance imaging found confluent white matter lesions throughout her brain. Lumbar puncture was positive for JCV DNA, and she passed away soon after diagnosis. Only one other case report of a patient developing PML while taking both azathioprine and prednisone for sarcoidosis exists. Patients with PML commonly experience cognitive disturbances, which can mimic more common disease processes, such as HE and infections. Our patient presented with these comorbid conditions, which delayed diagnosis. There are no established treatment options for PML patients without HIV except withdrawal of the offending medications. Clinicians must maintain awareness to diagnose PML as it has a broad presentation pattern, and comorbid conditions can further complicate diagnosis. Patients should be maintained on the minimally effective dose of immunosuppression to reduce the risk of PML development.

## Introduction

Progressive multifocal leukoencephalopathy (PML) is a rare, progressively fatal demyelinating neurologic disease caused by the John Cunningham virus (JCV), a ubiquitous polyomavirus [1]. While 82% of affected patients have human immunodeficiency virus (HIV), any immunosuppressing process is a significant risk factor for PML. Medications are a particularly important risk factor for PML development. Specifically, monoclonal antibodies, such as natalizumab, are the most prominent class of medications that are associated with PML development [2,3].

PML is very rare, with a variable incidence based on the predisposing disease process impacting patients. In a previous cohort of HIV-positive patients, the incidence of PML was reported at 0.06 cases per 100 patient-years since 1996, but before the expanded use of antiretroviral therapy for HIV, this cohort had an incidence of 0.24 cases per 100 patient-years [4]. Patients treated with natalizumab have an estimated incidence of 4.2 cases of PML per 1,000 patients, while bone marrow transplant patients have an incidence of 35.4 cases per 100,000 patient-years [5]. Interestingly, the JCV is extremely common, with estimates of up to more than 80% of the population having evidence of seropositivity for the virus and up to 40% of otherwise healthy adults with evidence of urinary shedding of the virus [5,6]. Of course, it is the state of immunosuppression that allows the virus to become clinically apparent in the form of PML [5]. Notably, after initial discovery, only 230 cases of PML were reported between the years 1958 and 1982, but the rate of PML increased 20-fold over the next decade with the AIDS epidemic [1].

Diagnosis of PML is made by either histopathological demonstration of JCV in brain tissue or by typical magnetic resonance imaging (MRI) findings and characteristic clinical features, along with JCV detection in the cerebrospinal fluid [1]. There are other important conditions to consider when diagnosing PML. One such condition is multiple sclerosis (MS). Unlike PML, MS impacts the optic nerve and spinal cord and follows a course of clinical attacks followed by recovery, but PML tends to continually worsen without clinical recovery [7]. Another important condition to consider is HIV encephalitis. MRI findings differ significantly between HIV encephalitis and PML, as HIV encephalitis shows diffusely symmetric, bilateral involvement with atrophy and tends to have sparing of the subcortical white matter. Conversely, MRI findings of PML patients tend to be lesions that are asymmetric and multifocal, and involve the subcortical white matter [1,7].

The hallmark of treatment for PML is revival of the cellular immune response [8]. This has become quite feasible in HIV-induced PML with the introduction of antiretroviral therapy, but it can be quite difficult in cases of malignancy-induced PML or when important medications, such as corticosteroids, are found to be the cause of PML [8]. Multiple classes of therapeutic options have been trialed in patients with PML. Immune checkpoint inhibitors have been used in PML patients with mixed results, and recombinant interleukins, particularly interleukin-7, have been shown to increase the T-cell immune response in PML patients; however, they have also been associated with significant side effects, including immune reconstitution inflammatory syndrome (IRIS) [8]. Finally, allogenic JCV-specific T‐cell transplantation has shown some efficacy against PML in a limited number of patients, but it is a highly technical process that cannot be routinely performed at most institutions [8]. The main complication associated with PML, which is frequently seen in patients with HIV, is IRIS, and this is described as a sudden increase in the immune response due to the treatment of the underlying immunocompromising condition that leads to a sudden worsening of the previously noted neurological deficits [1]. IRIS can range from a mild worsening of symptoms to a fatal outcome, as mortality can be up to 28% with IRIS [1].

Furthermore, diagnosing PML can be difficult as it can present in a wide variety of ways. Symptoms depend on which areas of the brain are involved in the demyelinating disease process and can include symptoms ranging from cognitive and behavioral changes to motor deficits [1,5]. Other notable symptoms that can be present include speech disturbances, seizures, and visual abnormalities, yet interestingly, it does not impact the optic nerve directly [5,7]. The diagnosis of PML can be further complicated by comorbid conditions that lead to encephalopathy, like cirrhosis. Here, we present a unique presentation of PML in a patient on immunosuppressive agents, which are less frequently associated with PML development that was further confounded by other more common etiologies of encephalopathy.

## Case presentation

A 63-year-old female patient with cryptogenic cirrhosis complicated by hepatocellular carcinoma, cytomegalovirus (CMV) viremia, type 2 diabetes mellitus, hypothyroidism, Sjogren's syndrome, and sarcoidosis presented with several months of intermittent confusion, generalized weakness, and progressive dysphagia. Family at bedside stated the patient had been intermittently hospitalized throughout the year and was progressively more debilitated. Over the past few weeks, she had cessation of almost all activities of daily living and was intermittently disoriented to her situations and surroundings. She had also been complaining of an increased difficulty swallowing solids and liquids over several weeks. Relevant medical history was significant for chronic use of azathioprine 50 mg daily and prednisone 20-40 mg daily (40 mg daily at presentation) for approximately 15 years for sarcoidosis and Sjogren’s syndrome.

On presentation, the patient was afebrile and hemodynamically stable. The initial physical exam was significant for suprapubic tenderness to palpation. Given that she intermittently followed commands, a comprehensive neurologic exam was limited. She was oriented to self but not to time, location, or situation. Pertinent laboratory values from initial presentation are shown in Table 1. Urinalysis showed 3+ bacteria. The Model for End-Stage Liver Disease-3.0 score was 20 at presentation. Computed tomography chest/abdomen/pelvis showed cirrhosis with a stable 4.1 cm hepatic lesion, and mild compression deformities of the superior endplates of T11-L1 were present, but no other acute abnormalities were noted.

**Table 1:** Pertinent laboratory values assessed at patient presentation This table demonstrates relevant laboratory values obtained in the initial workup for the patient's encephalopathy

Lab test	Lab value	Reference range
Sodium	132 mmol/L	136-145 mmol/L
Calcium	8.1 mg/dL	8.4-10.5 mg/dL
Creatinine	0.84 mg/dL	0.72-1.25 mg/dL
Glucose	249 mg/dL	70-99 mg/dL
Aspartate aminotransferase	85 units/L	10-50 units/L
Alanine aminotransferase	132 units/L	0-55 units/L
White blood cells	7.1 × 10^3^/μL	3.9-10.7 × 10^3^/μL
Hemoglobin	10.4 g/dL	11.8-16.0 g/dL
Platelets	156 × 10^3^/μL	135-371 × 10^3^/μL
International normalized ratio	2.0	1.0
Thyroid-stimulating hormone	0.104 μunits/mL	0.350-3.600 μunits/mL
Free thyroxine	0.96 ng/dL	0.70-1.37 ng/dL
Hemoglobin A1c	6.9%	<6.5%
Cytomegalovirus quantitative DNA	271 (previously 777) international units/mL	None detected
Human immunodeficiency virus P24 antigen + 1/2 antibody	Nonreactive	Nonreactive

Given the patient’s dysphagia, esophagogastroduodenoscopy was performed and showed grade I esophageal varices without evidence of bleeding, portal hypertensive gastropathy, and esophageal ulcers that were negative for CMV on biopsy. Initially, the most likely etiology of the patient’s altered mental status was hepatic encephalopathy (HE) precipitated by acute cystitis. There was no change in mentation despite several days of adequate treatment with ceftriaxone. Antibiotics were transitioned to doxycycline for vancomycin-resistant *Enterococcus faecium*, which was isolated from the urine culture. Notably, lactulose and rifaximin administration continued with an adequate number of bowel movements for HE concerns. The patient’s mentation continued to wax and wane, and repeat exams by the team demonstrated left upper extremity neglect and very weak (1/5) grip strength once the patient was able to intermittently follow commands for a complete neurologic exam. Further imaging was obtained given these neurologic findings.

**Figure 1:**
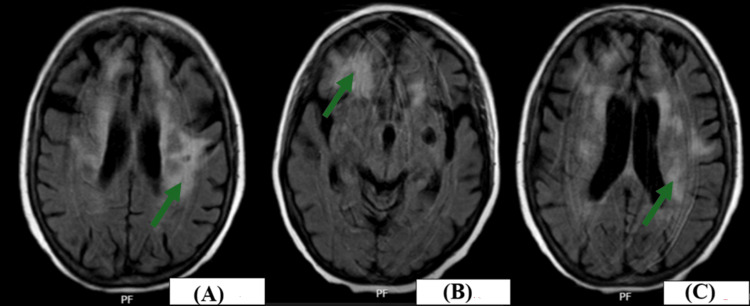
Confluent white matter lesions throughout all hemispheres seen on MRI brain FLAIR imaging (A,C) Left-sided cerebral periventricular white matter lesions (green arrows). (B) A right-sided frontal cortex white matter lesion (green arrow) MRI: magnetic resonance imaging; FLAIR: fluid-attenuated inversion recovery

MRI brain and cervical spine were obtained and were significant for confluent white matter lesions throughout the hemispheres on fluid-attenuated inversion recovery (FLAIR) phase imaging, and more focal increased confluent white matter abnormal FLAIR signal in the right frontal region. Multiple areas of bright signal on diffusion-weighted imaging without true restricted diffusion were noted, along with no intracranial enhancement. The cervical spine showed no gross abnormalities. Pertinent imaging is shown in Figure 1.

Lumbar puncture was subsequently obtained and cerebrospinal fluid (CSF) studies showed the following: 20 total cells (15% lymphocytes and 85% macrophages/monocytes), glucose 81 mg/dL, protein 59 mg/dL, IgG oligoclonal bands negative, cytology negative for malignancy, bacterial and fungal cultures with no growth, meningitis/encephalitis panel negative, and positive JCV polymerase chain reaction. Azathioprine was stopped, and a prednisone taper was initiated.

Despite these interventions, the patient continued to worsen from a mentation perspective and eventually refused all nutrition. Goals of care discussions occurred with the family, and inpatient hospice care was initiated. The patient passed away 10 days after the PML diagnosis.

## Discussion

PML is an overall very rare disease process. Case reports of PML development in patients without HIV are rare, and a review of the literature found only one other case report of a patient developing PML while being chronically immunosuppressed with azathioprine and prednisone for sarcoidosis [9]. As previously mentioned, certain medications, such as natalizumab, are more commonly associated with PML development; however, a review of 326 reported cases of drug-associated PML found one case with azathioprine monotherapy and 15 cases when glucocorticoids and azathioprine were administered together [10]. Our patient was chronically on this exact medication regimen of azathioprine and prednisone without cessation for decades. Appropriate medication review must be performed by clinicians at each encounter, and assessment for continued clinical indications for medications should also be maintained. While prednisone 20-40 mg daily is an appropriate treatment option for pulmonary sarcoidosis in the initial treatment phase of four to six weeks, tapering to 5-10 mg of prednisone daily must be performed after this phase to prevent systemic side effects, such as infections, including PML [11]. With the additional immunosuppressive effects of azathioprine, combined with her underlying chronic liver disease, this patient was at a very high risk of developing an opportunistic infection. This case underscores the crucial concept that patients must be maintained on the minimally effective dose of immunosuppression needed to control the underlying disease to limit infectious risks.

PML can be extremely difficult to diagnose for multiple reasons. As mentioned in the Introduction section, it is diagnosed by either histopathological demonstration of JCV in brain tissue or, as in the case of our patient, typical MRI findings and characteristic clinical features along with JCV detection in the CSF [1]. The typical lesion characteristics of PML on MRI are hyperintense on T2-weighted FLAIR sequences, involve subcortical and juxtacortical white matter, and are usually sharply delineated at the cortical border [1]. The diagnosis of PML is extremely important to make definitively in the clinical conundrum of encephalopathy, as autoimmune encephalitis was considered in this patient’s differential diagnosis, and high-dose steroid treatment was considered, which would have only led to the progressive worsening of her PML.

As noted in the Introduction section, symptoms experienced by patients with PML depend on which areas of the brain are involved in the demyelinating disease process and include cognitive and behavioral changes as well as motor deficits [1]. Our patient had profound left upper extremity weakness that could resemble other, much more common pathologies, such as a stroke. It is quite possible that this patient’s dysphagia was also related to her underlying PML [10]. Behavioral and cognitive changes have a broad toxic and metabolic differential diagnosis, and this includes many of the pathological processes that were considered and treated in this patient, such as infectious etiologies or HE [1]. When patients also present with these comorbid conditions, the diagnosis of PML can be extremely difficult to decipher.

PML recognition is important early in the disease process as it is a progressively fatal disease with no proven, established treatment options for patients without HIV other than immunosuppression cessation [1,12]. With the introduction of combination antiretroviral therapy, the one-year survival rate of PML in HIV positive patients increased from 10% to 50% [2]. Conversely, a previous study found the one-year mortality rate for non-HIV patients with PML was 60% as opposed to 25% for patients with HIV [13]. Overall, the increase in observed PML survival rates is believed to be due to the profound advances in HIV therapies [4]. Other than attempts at cessation of immunosuppressive medications, our patient did not have any viable treatment options for her PML, underscoring the importance of maintaining patients on the minimally effective dosage of immunosuppressive medications.

## Conclusions

A high index of clinical suspicion must be maintained to diagnose PML. It can present with a broad range of symptoms, especially if patients have comorbid medical conditions that can also independently lead to encephalopathy. In patients without HIV, medications, particularly immunosuppressive medications, should be strongly considered as the cause of PML. Furthermore, patients should be maintained on the minimally effective dose of immunosuppression, especially in the case of corticosteroids, to reduce the risk of developing infectious complications, such as PML.
